# Nitrogen Nutrition Promotes Rhizome Bud Outgrowth via Regulation of Cytokinin Biosynthesis Genes and an *Oryza longistaminata* Ortholog of *FINE CULM 1*

**DOI:** 10.3389/fpls.2021.670101

**Published:** 2021-04-30

**Authors:** Kyohei Shibasaki, Arika Takebayashi, Nobue Makita, Mikiko Kojima, Yumiko Takebayashi, Misato Kawai, Takushi Hachiya, Hitoshi Sakakibara

**Affiliations:** ^1^RIKEN Center for Sustainable Resource Science, Yokohama, Japan; ^2^Graduate School of Bioagricultural Sciences, Nagoya University, Nagoya, Japan; ^3^Department of Molecular and Functional Genomics, Interdisciplinary Center for Science Research, Shimane University, Matsue, Japan

**Keywords:** axillary bud outgrowth, cytokinin, nitrogen, *Oryza longistaminata*, rhizome

## Abstract

*Oryza longistaminata*, a wild rice, can propagate vegetatively via rhizome formation and, thereby, expand its territory through horizontal growth of branched rhizomes. The structural features of rhizomes are similar to those of aerial stems; however, the physiological roles of the two organs are different. Nitrogen nutrition is presumed to be linked to the vegetative propagation activity of rhizomes, but the regulation of rhizome growth in response to nitrogen nutrition and the underlying biological processes have not been well characterized. In this study, we analyzed rhizome axillary bud growth in response to nitrogen nutrition and examined the involvement of cytokinin-mediated regulation in the promotion of bud outgrowth in *O. longistaminata*. Our results showed that nitrogen nutrition sufficiency promoted rhizome bud outgrowth to form secondary rhizomes. In early stages of the response to nitrogen application, glutamine accumulated rapidly, two cytokinin biosynthesis genes, isopentenyltransferase, and *CYP735A*, were up-regulated with accompanying cytokinin accumulation, and expression of an ortholog of *FINE CULM1*, a negative regulator of axillary bud outgrowth, was severely repressed in rhizomes. These results suggest that, despite differences in physiological roles of these organs, the nitrogen-dependent outgrowth of rhizome axillary buds in *O. longistaminata* is regulated by a mechanism similar to that of shoot axillary buds in *O. sativa.* Our findings provide a clue for understanding how branched rhizome growth is regulated to enhance nutrient acquisition strategies.

## Introduction

Seed reproduction and vegetative reproduction are the two major modes of propagation in plants. Some perennial plant species that vegetatively reproduce expand their territories through stolon or rhizome growth (Guo et al., 2020). Rhizomes grow horizontally underground and form branches as secondary and tertiary rhizomes by the outgrowth of axillary buds that develop at rhizome nodes. Some rhizome tips developmentally transform into aerial green shoots and appear aboveground, becoming new ramets.

Wild rice is known for its substantial ecological diversity. *Oryza longistaminata*, an African wild rice species, is mainly propagated vegetatively via rhizome formation and proliferates vigorously (Vaughan, 1994). Both *O. longistaminata* and *Oryza sativa* contain the AA genome and have a high degree of genome synteny as revealed by genome sequencing (Reuscher et al., 2018; Li et al., 2020). *O. longistaminata* has also been used to study the genetic basis of rhizome development (Hu et al., 2011; Yoshida et al., 2016; Fan et al., 2017; Kyozuka, 2017; Bessho-Uehara et al., 2018; Toriba et al., 2020). The basic structural features of rhizomes are similar to those of aerial stems; rhizomes are composed of phytomer units consisting of a node, an internode, and an axillary bud (Yoshida et al., 2016). Crown roots develop at most of the rhizome nodes, suggesting that the branching growth of rhizomes is a strategy for efficient nutrition acquisition from the soil. Whereas rhizomes and aerial stems have similar anatomical features, the physiological features are different. Rhizomes are photosynthetic sink organs in which the internodes can elongate even during the vegetative stage of the parental ramet, whereas aerial stems are photosynthetic source organs in which internode elongation is tightly linked with the reproductive transition.

Studies of the molecular basis for shoot branching (tillering) in *O. sativa* show that three phytohormones, auxin, cytokinin, and strigolactone play key roles in regulating this developmental process (Beveridge and Kyozuka, 2010; Wang and Li, 2011; Gallavotti, 2013). Apically-derived auxin negatively regulates cytokinin biosynthesis by repressing *IPT4* and positively regulating strigolactone biosynthesis by *D10* (Arite et al., 2007; Minakuchi et al., 2010). Strigolactone induces the expression of *FC1* that negatively regulates axillary bud outgrowth, whereas expression of *FC1* is repressed by cytokinin (Minakuchi et al., 2010). As for nutritional factors that influence shoot branching in *O. sativa*, phosphate-starvation induces *de novo* synthesis of strigolactones that suppress shoot branching (Umehara et al., 2010). In contrast, nitrogen sufficiency up-regulates glutamine-mediated *IPT4* gene expression and plays a role in the promotion of shoot axillary bud outgrowth (Kamada-Nobusada et al., 2013; Ohashi et al., 2017; Sakakibara, 2021). A loss-of-function mutant of *glutamine synthetase1;2* (*GS1;2*) reduced the nitrogen-dependent induction of *IPT4* and the length of axillary buds, a phenotype that was rescued by the application of cytokinins (Ohashi et al., 2017). Nevertheless, how rhizome branching in *O. longistaminata* is regulated by nitrogen nutrition and the biological processes underlying rhizome axillary bud outgrowth remain to be elucidated.

In this study, we analyzed the growth of rhizome axillary buds in response to nitrogen nutrition and examined the involvement of cytokinin-mediated regulation in the promotion of bud outgrowth in *O. longistaminata*. Our results suggest that the nitrogen-dependent outgrowth of rhizome axillary buds in *O. longistaminata* is regulated by a mechanism similar to that for shoot axillary buds in *O. sativa*. This finding provides important insights for understanding the regulation of rhizome branching and growth in nutrient acquisition strategies.

## Results

### Effect of Nitrogen Nutrition on Rhizome Bud Outgrowth in *O. longistaminata*

**FIGURE 1:**
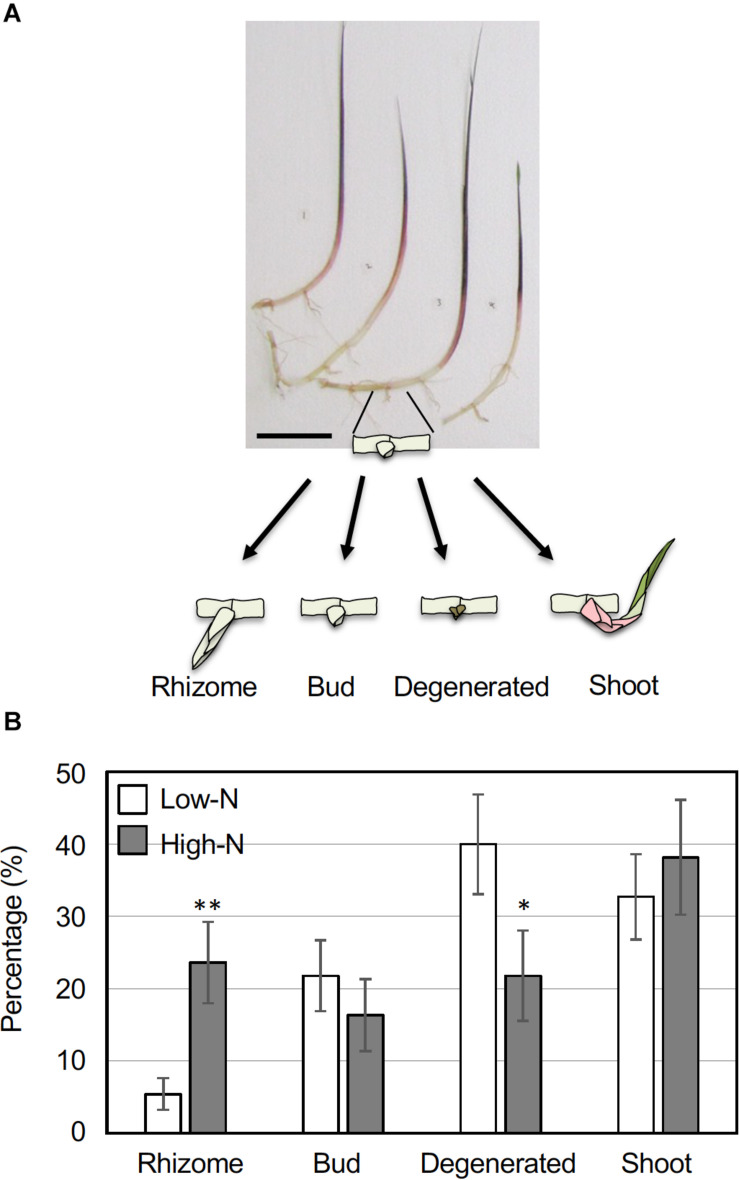
Growth response of *O. longistaminata* rhizome axillary buds to nitrogen nutrition. Young ramets having rhizomes at a similar growth stage were hydroponically grown in a low-N (200 μM NH_4_NO_3_) medium for 2 weeks. The ramets were subsequently transferred to either a low-N or high-N (2 mM NH_4_NO_3_) medium and incubated for an additional 2 weeks. Growth of the axillary buds on the rhizome nodes was monitored and classified into one of four categories: Rhizome (length > 10 mm), Buds (length < 10 mm), Degenerated, and Shoot. Detailed category definitions are explained in the text. **(A)** Typical ramets used in this experiment. Scale bar = 10 cm. **(B)** A comparison of rhizome growth in low-N and high-N media. Vertical bars represent the mean ± S.E. [*n* (ramet) = 13 for low-N, *n* = 14 for high-N]. Each ramet rhizome had 2 to 7 nodes, and the total number of nodes was 55 for low-N and 55 for high-N samples. *P < 0.05; **P < 0.01 (Student’s *t* test) compared to the low-N medium.

To examine the growth response of rhizome axillary buds to an inorganic nitrogen source, young ramets of comparable growth stages (Figure 1A) were hydroponically grown in a low-nitrogen (Low-N, 200 μM NH_4_NO_3_) medium for 2 weeks. The young ramets were further grown under a Low-N or a high-nitrogen (High-N, 2 mM NH_4_NO_3_) medium for another 2 weeks, and growth of the axillary buds was measured. The resulting growth status of the axillary buds was categorized into one of four patterns: secondary rhizomes whose internodes elongated greater than 1 cm (Rhizome), a bud that grew slightly but the length was less than 1 cm (Bud), a degenerated bud undergoing necrosis (Degenerated), and a developmentally transformed green shoot (Shoot; Figure 1A). The outgrowth of rhizome axillary buds was promoted under the High-N treatment with the percentage of secondary rhizomes significantly higher than in the Low-N treatment (Figure 1B). By contrast, the percentage of degenerated buds in the Low-N treatment was significantly higher than for the High-N treatment. In both nitrogen treatments, about 30 to 40% of the axillary buds became green shoots (Figure 1B). These results suggest that an inorganic nitrogen source promotes rhizome bud outgrowth to form secondary rhizomes.

### Phytohormone Profile in Rhizomes in Response to Nitrogen Application

**FIGURE 2:**
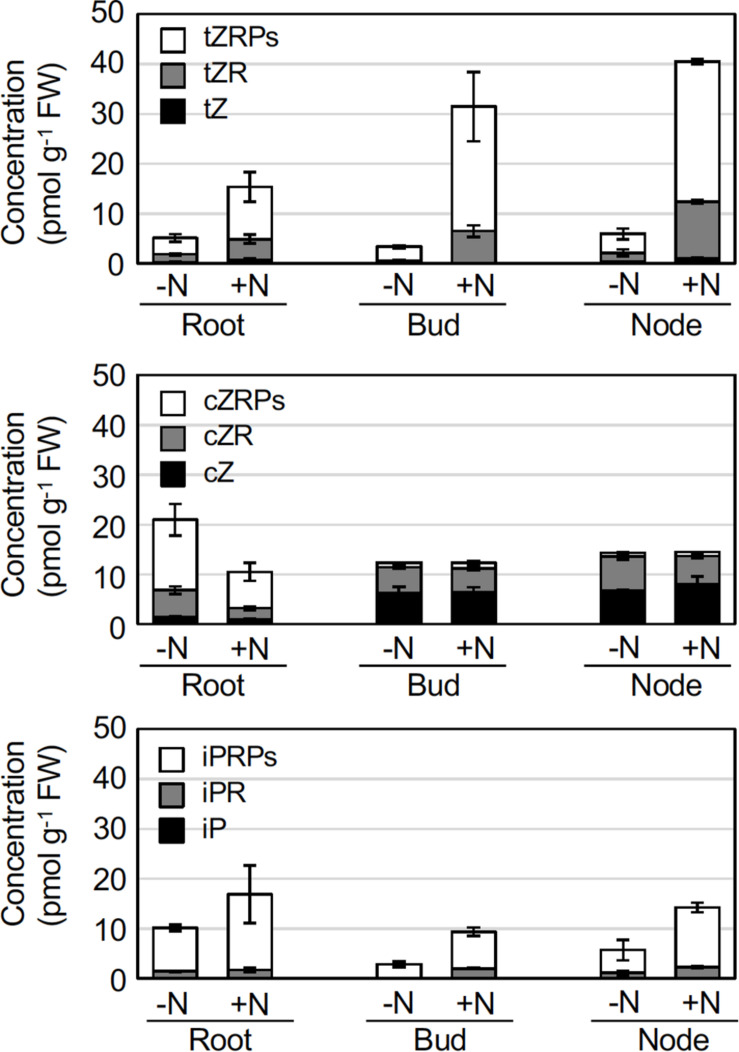
Effects of nitrogen applications on cytokinin concentrations in rhizome roots, buds, and nodes. Young ramets having rhizomes at a similar growth stage were hydroponically grown in a low-N (200 μM NH_4_NO_3_) medium for 2 weeks. The ramets were subsequently transferred to culture media containing no nitrogen (-N) or 2 mM NH_4_NO_3_ (+N). 6 h after transfer, rhizome roots, buds, and nodes were separately harvested, and the cytokinin hormone content was analyzed. Data are presented as the mean ± S.E. (*n* = 3). The complete data set is presented in Supplementary Table 1. tZ, *trans*-zeatin; tZR, tZ riboside; tZRPs, tZR 5’-phosphates; cZ, *cis*-zeatin; cZR, cZ riboside; cZRPs, cZR 5’-phosphates; iP, *N*^6^-(Δ^2^-isopentenyl)adenine; iPR, iP riboside; iPRPs, iPR 5’-phosphates; and FW, fresh weight.

In *O. sativa*, *de novo* cytokinin biosynthesis in roots and shoots is up-regulated by nitrogen sources, and cytokinins are involved in the promotion of axillary bud outgrowth of shoot stems (Kamada-Nobusada et al., 2013; Ohashi et al., 2017). To evaluate the effect of nitrogen application on the levels of phytohormones in rhizomes, we profiled the accumulation levels of the major phytohormones (cytokinins, ABA, and IAA) in crown roots, axillary buds, and nodes at 6 h after 2 mM NH_4_NO_3_ application. For the cytokinins, levels of *trans*-zeatin (tZ) and its precursors, such as tZ riboside and tZ riboside 5’-phosphates (tZRPs), and *N*^6^-(Δ^2^-isopentenyl)adenine riboside 5’-phosphates (iPRPs), precursors of iP, increased in crown roots, buds, and nodes of the NH_4_NO_3_ -treated rhizomes (Figure 2 and Supplementary Table 1). In contrast, the levels of other cytokinins and their precursors, such as cZ, cZR, and cZRPs, and most of the glucosides decreased after NH_4_NO_3_ application to the crown roots, but the levels of these compounds did not change in other plant organs (Figure 2 and Supplementary Table 1). Since tZRPs and iPRPs are initial products catalyzed by adenosine phosphate-isopentenyltransferase (IPT) and CYP735A (Sakakibara, 2006), these results suggest that *de novo* synthesis of cytokinins is activated by nitrogen application to the rhizomes. Interestingly, there was no significant difference in the levels of ABA or IAA resulting from supplemental NH_4_NO_3_ application to crown roots, axillary buds, or nodes (Supplementary Table 1).

To confirm a link between cytokinin accumulation and axillary bud outgrowth, we examined the effect of applying exogenous cytokinin on the rhizome. When we treated rhizomes with 1 μM tZ for 2 weeks, axillary bud growth was promoted (Supplementary Figure 1). These results support the hypothesis that cytokinin action is involved in nitrogen-dependent rhizome axillary bud outgrowth.

### Expression of Cytokinin Biosynthesis Genes in Response to Nitrogen Supply

**FIGURE 3:**
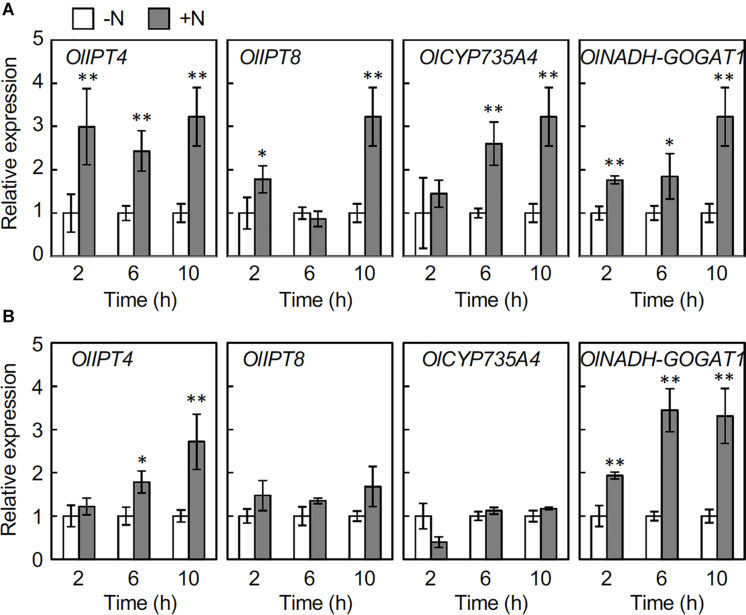
Changes in the expression of cytokinin biosynthesis genes in rhizomes responding to nitrogen application. Young ramets having rhizomes at a similar growth stage were hydroponically grown in a low-N (200 μM NH_4_NO_3_) medium for 2 weeks. The ramets were subsequently transferred to culture media containing no nitrogen (-N) or 2 mM NH_4_NO_3_ (+N). After incubation for the indicated times, rhizome roots and nodes with axillary buds were separately harvested. Total RNA prepared from the samples was subjected to RT-qPCR. Expression of the cytokinin biosynthesis genes in nodes including axillary buds **(A)** and roots **(B)**. Transcript abundance was normalized with *OlUBQ1* and expressed as relative values with respect to the value in the -N treatment. Values are means ± SE (*n* = 3). *P < 0.05; **P < 0.01 (Student’s *t* test) compared to the -N treatment.

To understand the underlying events leading to the nitrogen-dependent accumulation of cytokinins, we analyzed the expression of genes involved in cytokinin biosynthesis by reverse transcription-quantitative PCR (RT-qPCR). To specify the responsive genes, we selected orthologs of three cytokinin biosynthesis genes, *IPT, CYP735A*, and *LOG* from the *O. longistaminata* genomic database^1^ (Supplementary Table 2), and first analyzed the accumulation of the corresponding transcripts in rhizome nodes that include axillary buds at 6 h after the 2 mM NH_4_NO_3_ application (Supplementary Figure 2). Among the analyzed genes, the transcripts for *OlIPT4, OlIPT5, OlIPT8, and OlCYP735A4* accumulated in response to nitrogen application, suggesting that *de novo* cytokinin biosynthesis is up-regulated by nitrogen sources in the rhizome. *NADH-GOGAT1* that encodes a glutamate synthase was used as an indicator for the nitrogen response (Hirose et al., 1997). We further analyzed the expression of *OlIPT4, OlIPT8, and OlCYP735A4* in a time-course experiment. In the rhizome node that includes an axillary bud, the *OlIPT4* transcript accumulated within 2 h after 2 mM NH_4_NO_3_ application (Figure 3A). Transcripts for *OlIPT8* and *OlCYP735A4* also accumulated in response to nitrogen application but more slowly. In roots, the *OlIPT4* transcript accumulated gradually, but no clear induction of *OlIPT8* or *OlCYP735A4* was observed, although the expression of the nitrogen-responsive marker gene *OlNADH-GOGAT1* was induced (Figure 3B).

### Accumulation of Amino Acids in Response to the Nitrogen Supply

**FIGURE 4:**
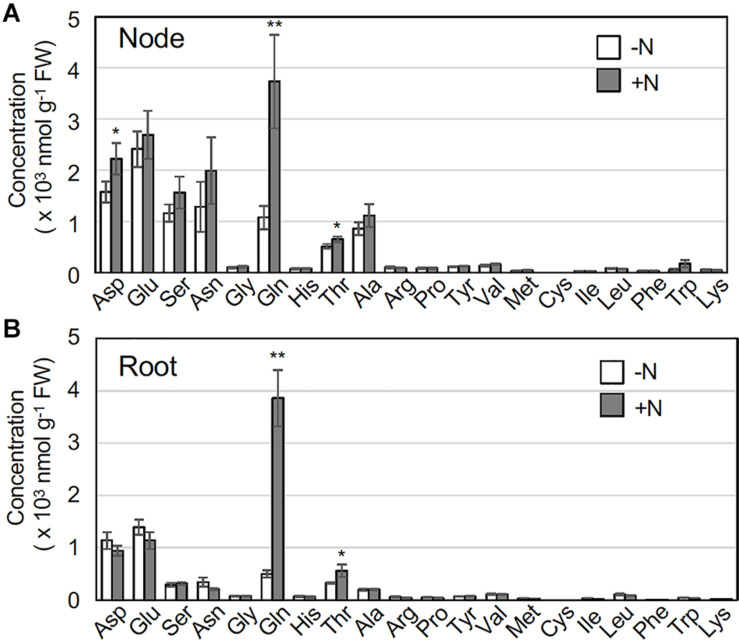
Effects of nitrogen application on amino acid concentrations in rhizome nodes and roots. Young ramets having rhizomes at a similar growth stage were hydroponically grown in a low-N (200 μM NH_4_NO_3_) medium for 2 weeks. The ramets were subsequently transferred to culture media containing no nitrogen (-N) or 2 mM NH_4_NO_3_ (+N) and incubated for 2 h. Rhizome nodes and roots were separately harvested and subjected to amino acid analysis. The amino acid concentrations in nodes including axillary buds **(A)** and roots **(B)**. Data are presented as the mean ± S.E. (*n* = 3). *P < 0.05; **P < 0.01 (Student’s *t* test) compared to the -N treatment. FW, fresh weight.

In *O. sativa*, a glutamine-related signal is a key indicator of *IPT4* gene induction (Kamada-Nobusada et al., 2013; Ohashi et al., 2017). To obtain insight into the relationship between glutamine and *IPT4* expression in *O. longistaminata*, we analyzed amino acid concentrations in rhizome nodes that include axillary buds and in roots in the presence or absence of supplemental nitrogen. Upon exposure to 2 mM NH_4_NO_3,_ glutamine accumulated significantly in both roots and nodes within 2 h (Figure 4). Although the difference was rather smaller, threonine also significantly accumulated in both organs. Aspartate levels in nodes were higher than those in roots, but no substantial differences in the levels of other amino acids were found between organs.

To obtain more direct evidence, we examined the effect of an exogenous application of glutamine on the expression of *OlIPT4*. When we treated rhizomes with 50 mM glutamine, *OlIPT4* expression in rhizome nodes was up-regulated in 6 h (Supplementary Figure 3), suggesting that glutamine-related signaling is involved in *OlIPT4* induction.

### Downregulation of *OlFC1* Expression in Response to the Nitrogen Supply

**FIGURE 5:**
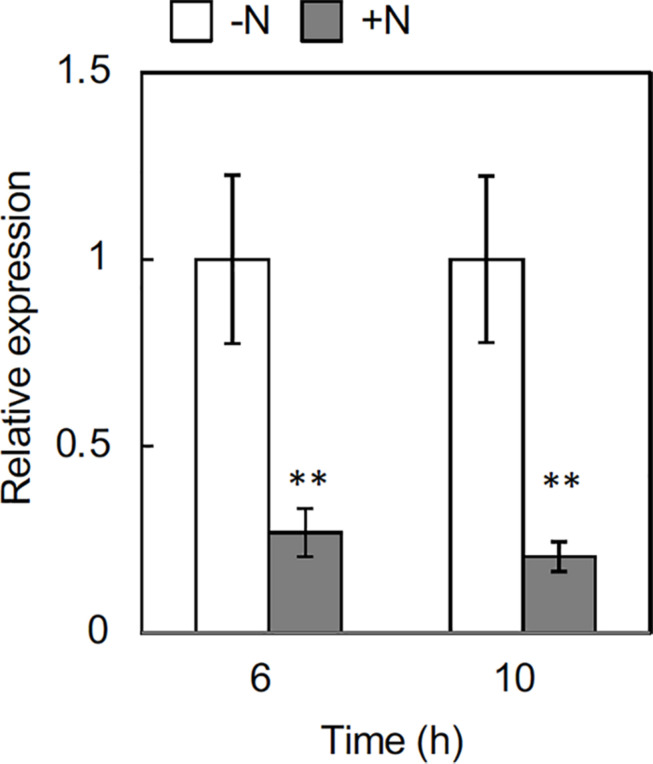
Effects of nitrogen application on the expression of *OlFC1* in rhizome buds. Young ramets having rhizomes at a similar growth stage were hydroponically grown in a low-N (200 μM NH_4_NO_3_) medium for 2 weeks. The ramets were subsequently transferred to culture media containing no nitrogen (-N) or 2 mM NH_4_NO_3_ (+N) and incubated for 6 h and 10 h. Total RNA prepared from the samples was subjected to RT-qPCR analysis. Transcript levels were normalized by *OlUBQ1* and expressed as relative values with respect to the value in the -N treatment. Values are the means ± SE (*n* = 3). **P < 0.01 (Student’s *t* test) compared to the -N treatment.

To further delineate the underlying processes regulating rhizome axillary bud outgrowth, we analyzed the effect of nitrogen supply on the expression of *OlFC1*, a key regulator of shoot axillary bud growth. Transcript accumulation markedly decreased in rhizome axillary buds in response to nitrogen supply at 6 h, and the repression continued at 10 h after treatment (Figure 5). This result suggests that nitrogen sufficiency down-regulates *OlFC1* expression.

## Discussion

In this study, we have shown that nitrogen nutrition promotes rhizome bud outgrowth to secondary rhizomes in *O. longistaminata*. The regulation of secondary rhizome formation in *O. longistaminata* relies on a mechanism similar to that for shoot axillary buds in *O. sativa*. In general, ample nitrogen nutrition promotes shoot growth but suppresses root system growth, with the opposite effect under nitrogen deficient conditions (Hermans et al., 2006; Gruber et al., 2013). Increasing the number of shoots (tillers) in response to nitrogen nutrition leads to larger amounts of aboveground biomass and more photosynthetic assimilation capacity. Also, prioritizing root system growth when nitrogen is deficient is a strategy for efficient nitrogen acquisition. In longer periods of *O. longistaminata* growth in the presence of different nitrogen treatments, growth of the secondary rhizomes was promoted and tertiary rhizomes branched under nitrogen-sufficient conditions (2 mM NH_4_NO_3_). Conversely, rhizome branching was suppressed and crown root growth was promoted under nitrogen-limiting conditions (200 μM NH_4_NO_3_; Supplementary Figure 4). Thus, the nitrogen-dependent promotion of rhizome elongation contributes to an expansion of the horizontal territory of vegetatively-propagated plants.

In the analysis of axillary bud growth patterns under Low-N and High-N treatments, there was no difference in the rate of differentiation to green shoots (Figure 1). Perhaps some of the axillary buds on rhizomes were already primed to become green shoots prior to the nitrogen treatment. Typically, the two basal nodes of ramets develop aerial shoot-type buds (Yoshida et al., 2016). The rate of axillary bud degeneration was higher in the low-N treatment. Deficiencies in the nitrogen supply could abort the growth of rhizome axillary buds in order to efficiently allocate nitrogen resources to a reduced number of rhizomes.

**FIGURE 6:**
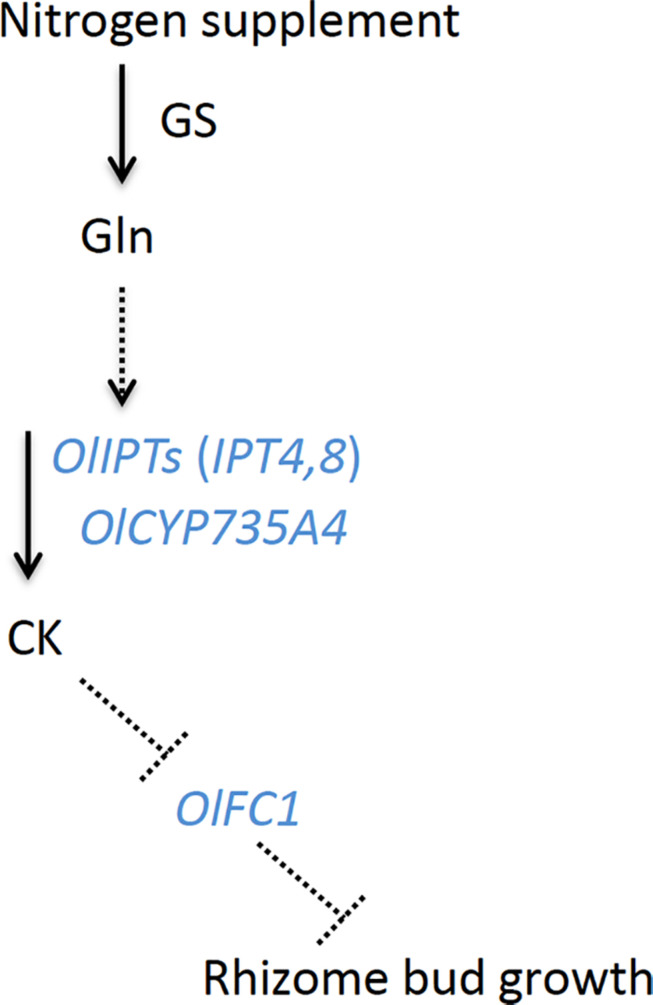
A model of hormonal control of rhizome axillary bud outgrowth in *O. longistaminata.* In this model, the nitrogen supply induces the accumulation of glutamine. Subsequently, a glutamine-related signal activates the expression of cytokinin biosynthesis genes, including *OlIPT4, OlIPT8*, and *OlCYP735A4*. The cytokinins negatively regulate the expression of *OlFC1*, the product of which functions to repress the outgrowth of the rhizome axillary bud. GS, glutamine synthetase; CK, cytokinin. Solid arrows represent the metabolic flow. Dashed arrows and T-end lines represent positive and negative regulation, respectively.

The expression of *OlIPT4* was up-regulated by supplemental nitrogen or exogenously applied glutamine (Figure 3 and Supplementary Figure 3). Also, the expression of *OlFC1* was repressed by an increased nitrogen supply (Figure 5). Studies on the regulation of shoot axillary bud outgrowth in *O. sativa* have shown that the expression of *FC1* is repressed by cytokinin (Minakuchi et al., 2010). Our findings in this study suggest a model for the nitrogen-dependent promotion of rhizome axillary bud outgrowth (Figure 6) that is similar to how shoot axillary bud outgrowth is regulated in *O. sativa*. A supply of inorganic nitrogen rapidly increases the concentration of glutamine and triggers the induction of *de novo* cytokinin biosynthesis via *OlIPT4* induction. The accumulated cytokinin suppresses the expression of *OlFC1*, thereby promoting outgrowth of the axillary bud.

The degree of nitrogen-dependent induction of *OlIPT4* and *OlCYP735A4* expression in the node was greater than that in the root (Figure 3) and corresponded with the changes in tZ-type cytokinin accumulation (Figure 2). The regulation of cytokinin biosynthesis gene expression in response to nitrogen nutrition in *O. sativa* has been primarily analyzed in roots but not in nodes. *IPT4* in *O. sativa* is mainly expressed in the phloem of vascular bundles (Kamada-Nobusada et al., 2013). *O. longistaminata* nodes might be a major organ for *de novo* cytokinin biosynthesis in response to nitrogen because nodes have a complex and dense vascular system (Yamaji and Ma, 2014).

*FC1* expression is induced by strigolactones in *O. sativa* (Minakuchi et al., 2010; Xu et al., 2015), whereas strigolactone biosynthesis, important for tiller growth, is induced by phosphorus deficiency (Umehara et al., 2010). The biosynthetic pathways and molecular species of strigolactones are very diverse among plant species. In maize, nitrogen deficiency strongly induces the biosynthesis of zealactone, a strigolactone (Ravazzolo et al., 2019). The regulation strigolactone biosynthesis in *O. longistaminata* has not yet been investigated. The involvement of strigolactones in axillary bud elongation of underground stems in response to nitrogen nutrition and the interplay between cytokinin and strigolactone signalings will be the subjects of future research.

## Materials and Methods

### Plant Materials and Growth Conditions

The perennial wild rice species *O. longistaminata* (IRGC10404) was used in this study. Plants were grown in soil in a temperature-controlled greenhouse with supplemental artificial light at 12 h light (30°C)/12 h dark (25°C) photoperiod. Young ramets of comparable growth stages (shoot lengths: 20 to 30 cm, rhizome lengths: 10 to 20 cm) were excised from parental ramets and grown for 2 weeks with aeration in a hydroponic culture solution (Kamachi et al., 1991) in which the NH_4_NO_3_ concentration was modified to either 200 μM NH_4_NO_3_ (low-N) or 2 mM NH_4_NO_3_ (high-N).

### Quantification of Phytohormones

Extraction and semi-purification of phytohormones from about 100 mg fresh weight of plant samples were performed as described previously (Kojima et al., 2009; Kojima and Sakakibara, 2012). Cytokinins were quantified using an ultra-performance liquid chromatography (UPLC)-tandem quadrupole mass spectrometer (ACQUITY UPLC^TM^ System/XEVO-TQS; Waters, Milford, MA, United States) with an octadecylsilyl (ODS) column (ACQUITY UPLC HSS T3, 1.8 μm, 2.1 mm × 100 mm, Waters; Kojima et al., 2009). ABA and IAA were quantified using an ultra-high performance liquid chromatography (UHPLC)-quadrupole-orbitrap mass spectrometer (UHPLC/Q-Exactive^TM^; Thermo Fisher Scientific, Waltham, MA, United States) with an ODS column (ACQUITY UPLC HSS T3, 1.8 μm, 2.1 mm × 100 mm; Waters; Kojima and Sakakibara, 2012; Shinozaki et al., 2015).

### Gene Cloning

Total RNA was prepared from plant samples using an RNeasy Plant Mini Kit with an RNase-free DNase Set (QIAGEN, Hilden, Germany). First-strand cDNA was synthesized by reverse transcription using a SuperScript^TM^ III First-Strand Synthesis System (Thermo Fisher Scientific) with oligo(dT)_12__–__18_ primers. cDNAs were amplified by PCR with specific primers and cloned into the pCR^TM^2.1-TOPO TA vector (Thermo Fisher Scientific).

### Quantitative PCR Analysis

Quantitative PCR (qPCR) analysis was performed using a StepOnePlus Real-Time PCR system (Applied Biosystems, Waltham, MA, United States) with a KAPA SYBR^®^ Fast qPCR Master Mix (2×) kit (KAPA Biosystems, London, United Kingdom). Transcript abundance was estimated using the relative quantification method (Livak and Schmittgen, 2001) with a *UBQ1* homolog as the internal standard for normalization. Gene locus IDs and the specific primers are listed in Supplementary Table 3.

### Determination of Free Amino Acids

Plant samples were powdered in liquid nitrogen and homogenized in 10 volumes of 10 mM HCl with 0.2 mM methionine sulfone as an internal control. The homogenate was centrifuged, and the supernatant was filtered through Ultrafree^®^ -MC filters (Merck Millipore, Burlington, MA, United States). Derivatization of amino acids was carried out using the Pico-Tag^®^ method (Waters), and the resulting derivatives were analyzed with an HPLC System (Alliance 2695 HPLC system/2475, Waters) using a Pico-Tag column as described in the manufacturer’s instruction manual.

## Data Availability Statement

The original contributions presented in the study are included in the article/Supplementary Material, further inquiries can be directed to the corresponding author/s.

## Author Contributions

KS and HS designed the research project. KS, AT, NM, MKa, MKo, YT, and TH conducted the research. KS, MKo, YT, and HS analyzed the data. KS and HS wrote the manuscript. All authors contributed to the article and approved the submitted version.

## Conflict of Interest

The authors declare that the research was conducted in the absence of any commercial or financial relationships that could be construed as a potential conflict of interest.

## Footnotes

1 http://olinfres.nig.ac.jp/
