# Supplementary material for: Nitrogen Nutrition Promotes Rhizome Bud Outgrowth via Regulation of Cytokinin Biosynthesis Genes and an Oryza longistaminata Ortholog of FINE CULM 1
Source: Front Plant Sci. 2021 Apr 30;12:670101. doi: 10.3389/fpls.2021.670101 (PMC8120282; doi:10.3389/fpls.2021.670101)
Supplement: Supplementary file 1 [file Data_Sheet_1.pdf]

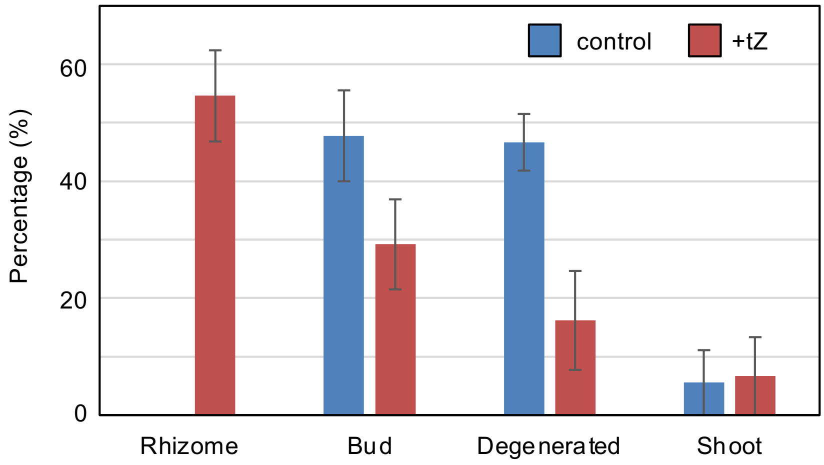

Supplementary Figure S1. Effects of cytokinin application on the growth of *O. longistaminata* axillary buds

Young ramets having rhizomes at a similar growth stage were hydroponically grown in a low-N (200 µM NH_4NO_3) medium for 2 weeks. The ramets were subsequently transferred to either a low-N medium (control) or low-N medium supplemented with 1 µM tZ (+tZ) and incubated for an additional 2 weeks. Growth of the axillary buds on the rhizome nodes was monitored. Detailed category definitions are defined in the legend to Figure 1. Vertical bars represent the mean ± S.E. [n (ramet) = 3 for the control and 3 for the +tZ-treated samples]. Each ramet rhizome had 3 to 7 nodes, and the total number of nodes was 17 for the control and 15 for the +tZ samples. There was no significant difference between the treated samples and the control as evaluated by Student's *t* test.

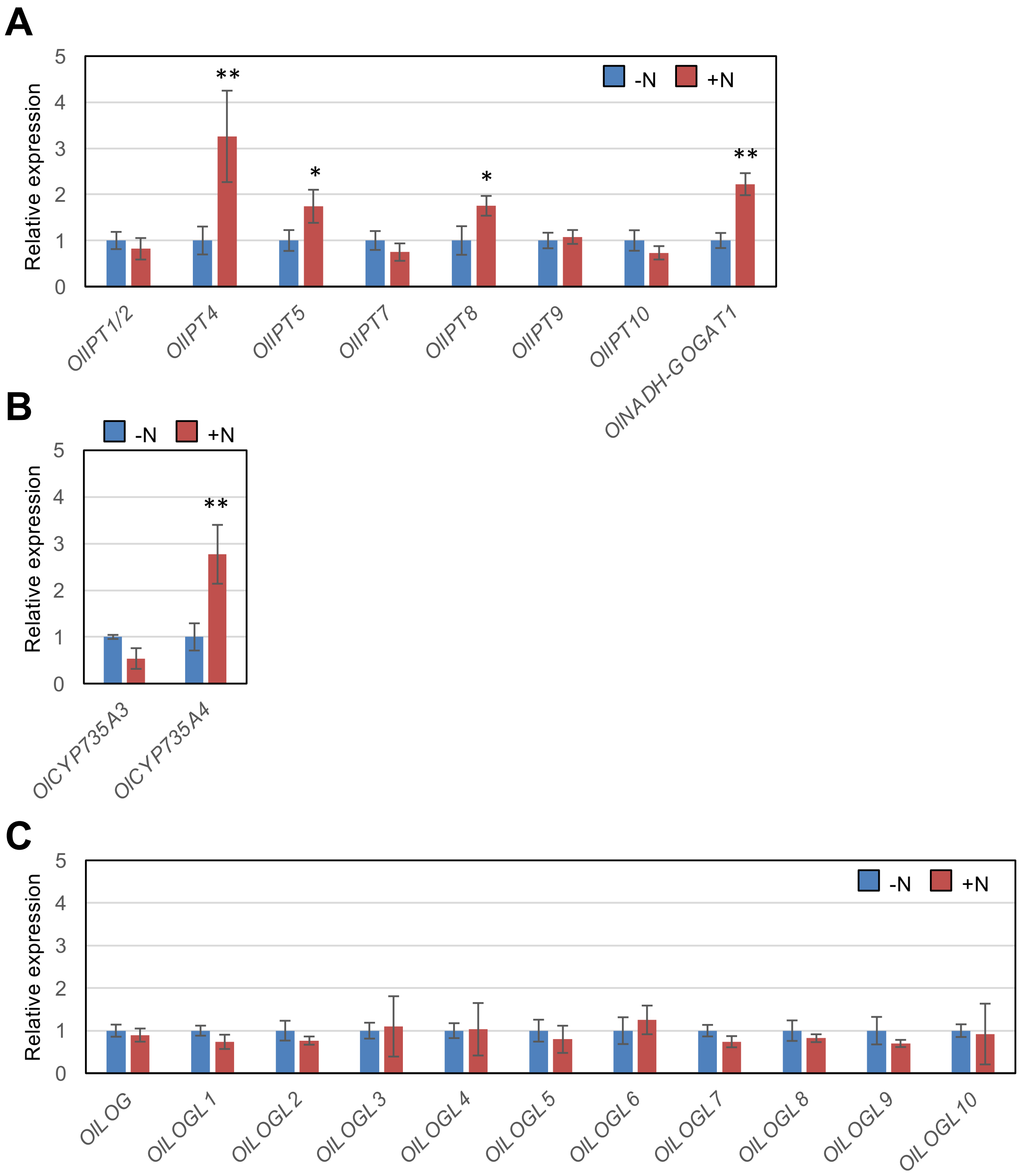

Supplementary Figure S2. Effects of nitrogen application on the expression of cytokinin biosynthesis genes in rhizome nodes

Young ramets having rhizomes at a similar growth stage were hydroponically grown in a low-N (200 μM NH_4NO_3) medium for 2 weeks. The ramets were subsequently transferred to culture media containing no nitrogen (-N) or 2 mM NH_4NO_3 (+N) and incubated for 6 h. Total RNA prepared from the samples was subjected to RT-qPCR analysis to monitor the relative expression of *IPTs* **(A)**, *CYP735As* **(B)**, and *LOGs* **(C)**. Transcript abundance was normalized with *OlUBQ1* and expressed as relative values with respect to the value in the -N treatment. Data are presented as the mean ± S.E. (n = 4 for -N, n = 3 for +N). *$P < 0.05$; **$P < 0.01$ (Student's *t* test) compared to the -N treatment.

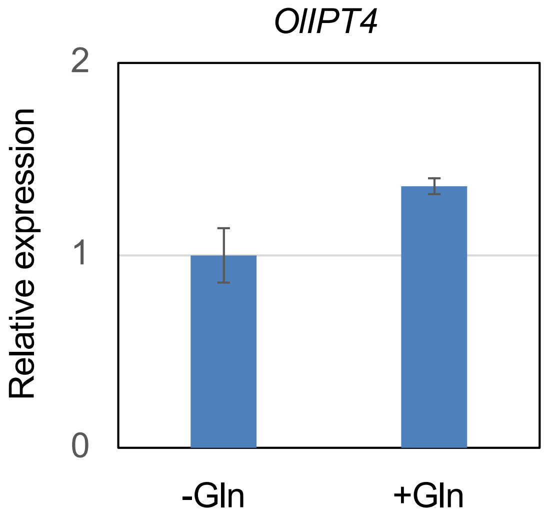

Supplementary Figure S3. Effects of glutamine application on the expression of *OlIPT4* in rhizome nodes

Young ramets having rhizomes at a similar growth stage were hydroponically grown in a low-N (200 μM NH_4NO_3) medium for 2 weeks. The ramets were subsequently transferred to the culture media containing no glutamine (-Gln) or 50 mM Gln (+Gln) and incubated for 6 h. Total RNA prepared from the samples was subjected to RT-qPCR analysis. Transcript abundance was normalized with *OlUBQ1* and expressed as relative values with respect to the value in the -Gln treatment. Data are presented as the mean ± S.E. (n = 3). There was no significant difference between the -Gln and +Gln treatments as evaluated by Student's *t* test.

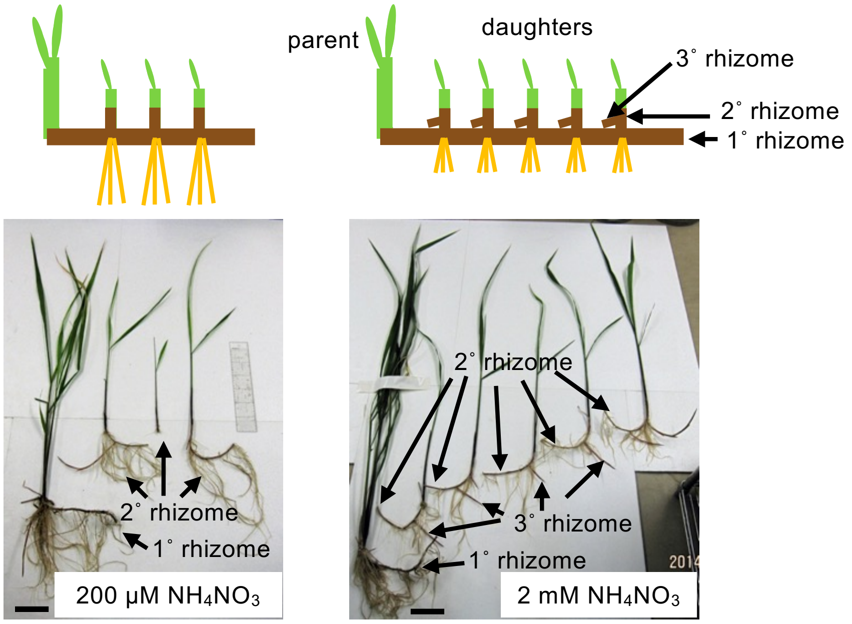

Supplementary Figure S4. Nitrogen concentration effects on the growth response of *O. longistaminata* rhizomes

Young ramets having rhizomes at a similar growth stage were hydroponically grown in 200 μM NH_4NO_3 or 2 mM NH_4NO_3 for 8 weeks. The culture media were renewed every week. At the end of the 8-week period, each ramet was excised and photographed. 1˚ rhizome, primary rhizome grown from the parent ramet; 2˚ rhizome, secondary rhizome developed on the node of the primary rhizome; 3˚ rhizome, tertiary rhizome developed on the node of the secondary rhizome. Scale bars = 10 cm.

Supplementary Table 1. Hormone concentrations in rhizome roots, buds and nodes exposed to low or high nitrogen sources

Hormone species	Root			Bud			Node		
	Low-N	High-N		Low-N	High-N		Low-N	High-N	
	pmol g^{-1} fresh weight			pmol g^{-1} fresh weight			pmol g^{-1} fresh weight		
tZ	0.25 ± 0.11	0.70 ± 0.23		B.Q.	B.Q.		0.35 ± 0.07	1.01 ± 0.09	**
tZR	1.57 ± 0.19	4.20 ± 0.89		0.52 ± 0.13	6.49 ± 1.17	*	1.78 ± 0.65	11.36 ± 0.34	**
tZRPs	3.31 ± 0.75	10.43 ± 2.97		2.80 ± 0.28	24.96 ± 6.97		3.80 ± 1.08	28.12 ± 0.55	**
cZ	1.48 ± 0.08	0.99 ± 0.16		6.13 ± 1.34	6.36 ± 1.01		6.65 ± 0.15	8.04 ± 1.51	
cZR	5.29 ± 0.78	2.19 ± 0.37	*	5.36 ± 0.39	4.78 ± 0.34		6.90 ± 0.63	5.63 ± 0.45	
cZRPs	14.16 ± 3.21	7.29 ± 1.79		0.81 ± 0.10	1.24 ± 0.31		0.82 ± 0.05	0.72 ± 0.07	
DZ	B.Q.	B.Q.		B.Q.	B.Q.		0.28 ± 0.04	0.35 ± 0.12	
DZR	B.Q.	B.Q.		B.Q.	0.22 ± 0.01		0.34 ± 0.03	0.44 ± 0.16	
DZRPs	B.Q.	B.Q.		B.Q.	B.Q.		0.06 ± 0.02	0.12 ± 0.02	
iP	0.08 ± 0.02	0.04 ± 0.01		B.Q.	B.Q.		0.09 ± 0.03	0.09 ± 0.01	
iPR	1.34 ± 0.11	1.65 ± 0.45		B.Q.	1.98 ± 0.13		0.96 ± 0.40	2.15 ± 0.15	
iPRPs	8.69 ± 0.62	15.18 ± 5.81		2.80 ± 0.58	7.37 ± 0.82	*	4.63 ± 2.04	11.99 ± 0.97	
tZ7G	B.Q.	B.Q.		B.Q.	B.Q.		B.Q.	B.Q.	
tZ9G	7.52 ± 1.11	3.38 ± 0.78	*	24.98 ± 5.21	38.86 ± 12.64		38.24 ± 10.74	41.11 ± 7.12	
tZOG	0.22 ± 0.03	B.Q.		2.53 ± 0.01	1.98 ± 0.02	*	0.83 ± 0.07	0.87 ± 0.05	
cZOG	84.07 ± 6.11	36.89 ± 9.10	*	693.86 ± 9.75	757.61 ± 46.73		515.74 ± 12.79	537.82 ± 14.51	
tZROG	0.12 ± 0.02	B.Q.		B.Q.	B.Q.		0.37 ± 0.04	0.36 ± 0.02	
cZROG	44.22 ± 2.40	25.48 ± 1.45	**	132.43 ± 10.20	153.03 ± 0.37		78.82 ± 4.00	74.68 ± 1.98	
tZRPsOG	B.Q.	B.Q.		B.Q.	B.Q.		B.Q.	B.Q.	
cZRPsOG	6.27 ± 1.53	3.10 ± 0.56		11.80 ± 2.02	13.86 ± 1.20		8.74 ± 1.55	7.65 ± 0.46	
DZ9G	B.Q.	B.Q.		B.Q.	B.Q.		B.Q.	B.Q.	
iP7G	B.Q.	B.Q.		B.Q.	B.Q.		B.Q.	B.Q.	
iP9G	1.96 ± 0.06	1.33 ± 0.14	*	0.69 ± 0.09	0.55 ± 0.05		2.03 ± 0.45	1.68 ± 0.17	
ABA	10.81 ± 1.28	12.94 ± 0.25		72.46 ± 13.73	70.21 ± 2.77		37.88 ± 6.35	46.73 ± 0.92	
IAA	99.52 ± 20.44	82.89 ± 14.13		209.03 ± 35.82	217.21 ± 88.02		72.88 ± 2.32	75.66 ± 2.31	
IAAsp	B.Q.	B.Q.		B.Q.	B.Q.		B.Q.	B.Q.	

Young ramets having rhizomes at a similar growth stage were hydroponically grown in a low-N (200 μM NH_4NO_3) medium for 2 weeks. The ramets were subsequently transferred to the low-N or high-N (2 mM NH_4NO_3) media and incubated for 6 h. Rhizome roots, buds, and nodes were separately harvested for hormone analysis. Data are presented as the mean ± S.E. (n = 3). Asterisks indicate significant differences compared to the low-N samples in Student's *t* test. *, $P < 0.1$; **, $P < 0.05$; ***, $P < 0.01$ (n = 3). tZ, *trans*-zeatin; tZR, tZ riboside; tZRPs, tZR 5'-phosphates; cZ, *cis*-zeatin; cZR, cZ riboside; cZRPs, cZR 5'-phosphates; DZ, dihydrozeatin; DZR, DZ riboside; DZRPs, DZR 5'-phosphates; iP, N^6-(Δ^2-isopentenyl)adenine; iPR, iP riboside; iPRPs, iPR 5'-monophosphate; tZ7G, tZ-7-*N*-glucoside; tZ9G, tZ-9-*N*-glucoside; tZOG, tZ-*O*-glucoside; cZOG, cZ-O-glucoside; tZROG, tZR-*O*-glucoside; cZROG, cZR-*O*-glucoside; tZRPsOG, tZRPs-*O*-glucoside; cZRPsOG, cZRPs-*O*-glucoside; iP7G, iP-7-*N*-glucoside; iP9G, iP-9-*N*-glucoside. ABA, abscisic acid; IAA, indole-3-acetic acid; IA-Asp, indole-3-acetyl-aspartic acid. B.Q., below the quantification limit.

Supplementary Table S2. Gene correspondence between *O. sativa* and *O. longistaminata*

Gene name in *O.s.*	Locus ID (RAP-DB)	Gene name in *O.l.*	Locus ID
OsIPT1	Os03g0358900	*OlIPT1/2*	OL03G001671
OsIPT2	Os03g0356900	*OlIPT1/2*	OL03G001671
OsIPT3	Os05g0311801		*
OsIPT4	Os03g0810100	*OlIPT4*	OL03G003563
OsIPT5	Os07g0211700	*OlIPT5*	OL07G000658
OsIPT6	Os07g0190150		**
OsIPT7	Os05g0551700	*OlIPT7*	OL05G002334
OsIPT8	Os01g0688300	*OlIPT8*	OL01G002653
OsIPT9	Os01g0968700	*OlIPT9*	OL01G004502
OsIPT10	Os06g0729800	*OlIPT10*	OL06G002644
CYP735A3	Os08g0429800	*OlCYP735A3*	OL08G001277
CYP735A4	Os09g0403300	*OlCYP735A4*	OL09G000777
LOG	Os01g0588900	*OlLOG*	OL01G002062
LOGL1	Os01g0708500	*OlLOGL1*	OL01G002786
LOGL2	Os02g0628000	*OlLOGL2*	OL02G002291
LOGL3	Os03g0109300	*OlLOGL3*	OL03G000052
LOGL4	Os03g0697200	*OlLOGL4*	OL03G002759
LOGL5	Os03g0857900	*OlLOGL5*	OL03G003882
LOGL6	Os04g0518800	*OlLOGL6*	OL04G001718
LOGL7	Os05g0541200	*OlLOGL7*	OL05G002268
LOGL8	Os05g0591600	*OlLOGL8*	OL05G002619
LOGL9	Os09g0547500	*OlLOGL9*	OL09G001632
LOGL10	Os10g0479500	*OlLOGL10*	OL10G001109
FC1	Os03g0706500	*OlFC1*	OL03G002830
NADH-GOGAT1	Os01g0681900	*OlNADH-GOGAT1*	OL01G002614
UBQ1	Os03g0234200	*OlUBQ1*	OL03G000950

*The *O. longistaminata* gene corresponding to *OsIPT3* was not annotated in the *O. longistaminata* genomic database.

**The genomic region including the locus corresponding to *OsIPT6* was in unmapped contigs.

Supplementary Table 3. Primers used in this study

Gene name	Locus ID	Forward (5' to 3')	Reverse (5' to 3')
OlIPT1/2	OL03G001671	ACCAAGCCCAAGGTTATCTTCGTGC	TCGTCGGTGACCTTGTTGGTGATGA
OlIPT4	OL03G003563	GCCGTCCGGTCATTCCTCTT	AGACGGAGATGTTGCCAGAAG
OlIPT5	OL07G000658	CCAATGCCATCGAGGACATCAA	GCGGTCTAGCCTCACGATCT
OlIPT7	OL05G002334	TCTGGGTCCACGTCGATGAG	CGGTTGTCGTGGCGAAGTAC
OlIPT8	OL01G002653	ATGGTGGAGGAGCTCGAGGA	TGATCTCGTCTATCGCCGCG
OlIPT9	OL01G004502	AGGGTCGACTGCATGATTGATGA	GCCGCAGCCCTTGGGTATAA
OlIPT10	OL06G002644	CCACCGACATCTCACCGATCA	TGTGTCGGCCAGCATCTCTT
OlCYP735A3	OL08G001277	CGGTGTCAGTGTCGATCCGA	ACGTCCTCTGGTCCAACACGTA
OlCYP735A4	OL09G000777	CGAGCAAGTACAGGAGGGAGA	CGACAGAAGCATAGCCAGCA
OlLOG	OL01G002062	CGGTGGTCGCCATGTCATTG	CAGCAAACCGGGCCATTTCA
OlLOGL1	OL01G002786	TCGCGCAGACGGTTCTTGAT	GGCGTGCCATCTCAGCTTTC
OlLOGL2	OL02G002291	AACCGGTTGGGCTGCTCAAT	GCGGGCACTAGGTCTGATGA
OlLOGL3	OL03G000052	GCTCGCCGCATCATCGTATC	AGCCCAGACGCAACCCTATC
OlLOGL4	OL03G002759	ACGTTGGGAGAGGCGAAAGT	ACCTGGCAGGGCGATGAAAG
OlLOGL5	OL03G003882	CGGCGAACAACGATAGCAAGAA	CACCTGGTACGACGCCTTCT
OlLOGL6	OL04G001718	TGACAAGGTCGTGCCGAAGA	TGGTCGCCTTGTTGCCTTCT
OlLOGL7	OL05G002268	TGCTCGCGTTGTTCGACAAA	TCCTGGTGCAACCGAGTGTA
OlLOGL8	OL05G002619	TGGTGGAGAGGGGCATAGAC	ATGGCGACCTCCAGCATGAA
OlLOGL9	OL09G001632	AGGAGGTGGTGGAGGTGATC	GACGGCCTTGTCGACGAAAG
OlLOGL10	OL10G001109	CAGCAGCCAGGGGAAGAAGA	CCGCCGTACACCAGGTCTAT
OlFC1	OL03G002830	GGACATACCGCTTTACCAACAG	GTTGCTGTGCTGCTGCTGCTT
OlNADH-GOGAT1	OL01G002614	CAGCTGTCGACAAGTACTTGTC	CCAAAATAACTGTACATTATT
OlUBQ1	OL03G000950	GCCGGGTTCATGGACTGGTTA	GGTTCAACAACATCCAGGGAGAT
